# Numerical Investigation of Special Heat Transfer Phenomenon in Wire-Wrapped Fuel Rod of SFR

**DOI:** 10.3390/mi13060935

**Published:** 2022-06-11

**Authors:** Xuefeng Tan, Bing Wang, Yun Guo, Miao Hu

**Affiliations:** 1 School of Nuclear Science and Technology, University of Science and Technology of China, Hefei 230026, China; dkdmy@mail.ustc.edu.cn (X.T.); guoyun79@ustc.edu.cn (Y.G.); 2 China Ship Development and Design Center, Wuhan 430064, China; wb14he@mail.ustc.edu.cn; 3 Department of Chemical and Materials Engineering, Hefei University, Hefei 230601, China

**Keywords:** wire-wrapped fuel bundle, heat transfer, SFR, microchannel

## Abstract

Sodium-cooled reactors (SFR) have always been recognized as one of the most promising candidates for the fourth-generation nuclear systems as announced by the Generation-IV International Forum. In the design of SFR, helical wire-wrapped rod is applied to stabilize the structure of the rod bundle and enhance coolant mixing. Although there has been considerable research on SFR in computational fluid dynamics (CFD), the phenomenon of heat transfer has rarely been paid attention to. This article discovered that there exists reversed heat flux from coolant to wrapped wire, which is contrary to our usual understanding. This phenomenon has not been reported in previous CFD calculations. Hence, a solid heat conduction model is proposed to prove this phenomenon and analyze the heat transfer process. The simulation results show that the wrapping wire embedding depth, the shape of the calculation domain and the physical properties of all components have great influence on the magnitude of the reversed heat flux. The present findings will have strong influence on the temperature field and maximum value of the fuel rod as well as profound reference value for future flow calculation, especially in grid generation and treatment of the junction between the winding wire and fuel rod.

## 1. Introduction

It has been widely acknowledged that fast reactors have a higher neutron utilization compared to thermal reactors. So far, historical experiences of design, construction, testing, operation, inspection and repair of past or existing demonstration and/or prototype SFRs, such as PFR (UKAEA), BN-350 (Kazakhstan), BN-600 (Russia) and Monju (Japan), have established the technological basis of the SFR [1]. Wire-wrapped pins have been presented as a solution to enhance heat transfer in the sub-channel between the fuel pins due to the small core geometry and higher heat flux compared with PWR; the general idea is that a helical wire wrapping forces the fluid to rotate around the fuel pins [2]. The flow and heat transfer are a long-standing research topic owing to the special constructure. Much related numerical and experimental research has been done to help understand the effects of wire-wrapping on the fuel pin.

Novendstern developed a semi-empirical model to predict pressure drop in a wire-wrapped fuel pin bundle, which is able to predict the value of the pressure drop within 14% over a wide range of geometries in the turbulent flow regime [3]. Gajapathy et al. analyzed the sodium flow and temperature distributions in heat generating fuel pin bundles with helical spacer wires by using a commercial CFD code, in which 7, 19 and 37 fuel pin bundles were taken into accountant. It was found that normalized outlet velocities were nearly equal to unity, and this is in great agreement with published hydraulic experimental measurements [4]. Hamman and Berry simulated three-dimensional flow distributions of sodium in the sub-channels of a 19-pin fuel bundle for large-scale problems, in which the results have a difference of 9–15% in comparison with some correlations [5]. Volkov et al. found that using high-Reynolds k-ε turbulence model to simulate the flow in the fuel pin bundle could reduce the dimension of the computational mesh. The results are in good agreement with the empirical dependences and international calculations [6]. Talebi et al. conducted a parameter research of the Reynolds number, inlet temperature and heat flux on the fluid flow, and heat transfer inside the sub-channel with a CFD code that solves the algorithm [7]. The results show that the wire-wrapped spacers created a uniform fluid velocity and temperature in the cross-section of the channel.

Tao Wu et al. conducted numerical research on microchannel heat sinks under high temperature conditions for diverse cross-section shapes, different liquid metal and various inlet velocities [8]. It was found that lithium and circle are the most appropriate choices for working fluid and microchannel cross-section shape. In addition, the inlet velocities has a significant influence on the pressure drop and heat transfer. Ying et al. analyzed different dimples geometries of heat exchanger channel numerically and experimentally to find that hemispherical dimple channels have the highest heat transfer coefficient and present better overall thermal performance [9].

Bovati et al. used Reynolds-Averaged Navier-Stokes (RANS) models to predict the axial and transverse pressure drops for a range of Reynolds numbers from 1270 to 100,000 in the numerical investigation of the 61-pin wire-wrapped bundle. They demonstrated that RANS is a suitable approach in predicting velocity and pressure fields in wire-wrapped rod bundles, with a relatively low computational effort [10].

Jeong and Song used an SST turbulence model to conduct a numerical investigation of a JAEA 7-pin fuel assembly experiment with local blockage for SFR. They compared the results with the experimental data and found small-scale vortex structures significantly enhance the convective heat transfer while large-scale vortex structures supply thermal energy near the heated cladding wall surface [11].

From all the above summaries, the conclusion can be drawn that, previous researches focused more on the fluid field because of the complex geometry, and so some details in heat transfer and temperature were ignored. At the same time, the grids or meshes near the wrapping wire are always sacrificed or compromised for the flow field calculations for CFD analysis. The most common method is to embed the winding wire into the fuel rod. Obviously, it will have a strong influence on the local heat transfer. This article discovered a phenomenon that reversed heat fluxes exist in the junction between the wrapping wire and the fuel rod which do not appear to have been reported. Hence, a solid model of heat transfer is proposed to prove this phenomenon and explore the factors which may have influence on it.

## 2. Reversed Heat Fluxes in Fluid Flow

As Merzari et al. [12] reported in 2012, two equation turbulence models are sufficient to predict turbulent properties governed by the tight lattice rod bundle and wire-wrapped spacer, and to estimate turbulent heat flux in liquid sodium. Employing a temperature-dependent property, the system of time-averaged governing equations for mass, momentum, and energy are given by:

(1) $$\frac{\partial \rho U_{j}}{\partial x_{j}}=0\;$$

(2) $$\frac{\partial \left(\rho U_{i}U_{j}\right)}{\partial x_{j}}=-\frac{\partial P}{\partial x_{i}}+\frac{\partial }{\partial x_{j}}\left(\mu_{eff}\frac{\partial U_{i}}{\partial x_{j}}\right)-\rho g_{i}$$

(3) $$\frac{\partial \left(\rho U_{j}T\right)}{\partial x_{j}}=\frac{\partial }{\partial x_{j}}\left(\left(\frac{\mu }{Pr}+\frac{\mu_{t}}{Pr_{t}}\right)\frac{\partial T}{\partial x_{j}}\right)$$

where U, T and P are velocity, temperature and pressure. ${\mathrm{Pr}}_{\;}$and ${\mathrm{Pr}}_{t}$ are Prandtl and turbulent Prandtl number, respectively. In this article, the k−ε model was selected as turbulence model, and more details about the wall treatment can be found in user guide of Star CCM+ [13]. Kays proposed a model to evaluate the value of turbulent Prandtl number as follows:

(4) $${\mathrm{Pr}}_{t}=0.85+\frac{0.7}{{\mathrm{Pe}}_{t}}$$

The initial turbulent intensity I is set to 5% which is a default value of Star CCM+. Based on turbulent intensity, turbulent kinetic energy is evaluated by $k=\frac{3}{2}{\left(I\bar{U}\right)}^{2},\;$ in which $\bar{U}$ is the averaged coolant velocity and hydraulic diameter of rod bundle, and is chosen as the characteristic length.

### 2.1. Geometry Model of Fluid Flow

**Figure 1:**
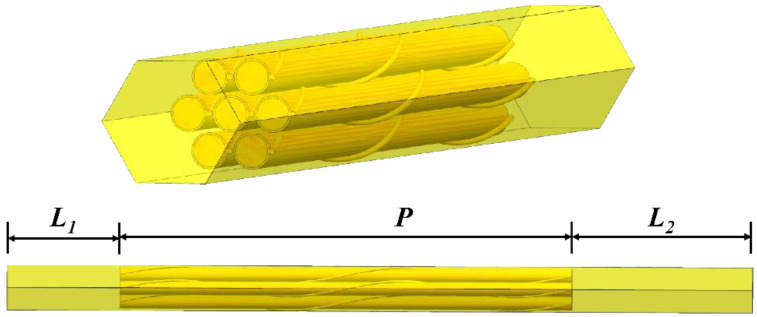
The calculation domain of 7-pin fuel bundle with inlet and outlet sections.

**Figure 2:**
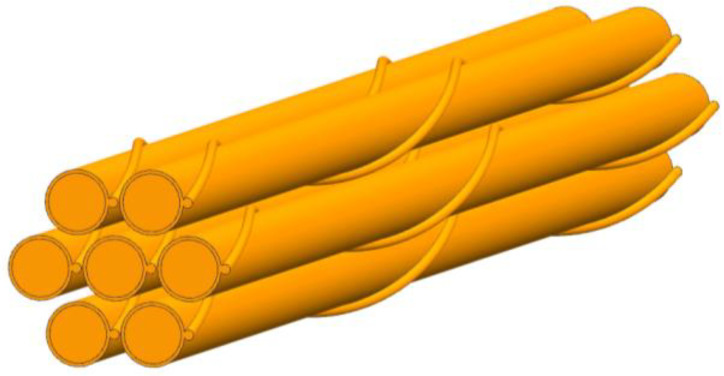
The wire-wrapped fuel bundle.

**Figure 3:**
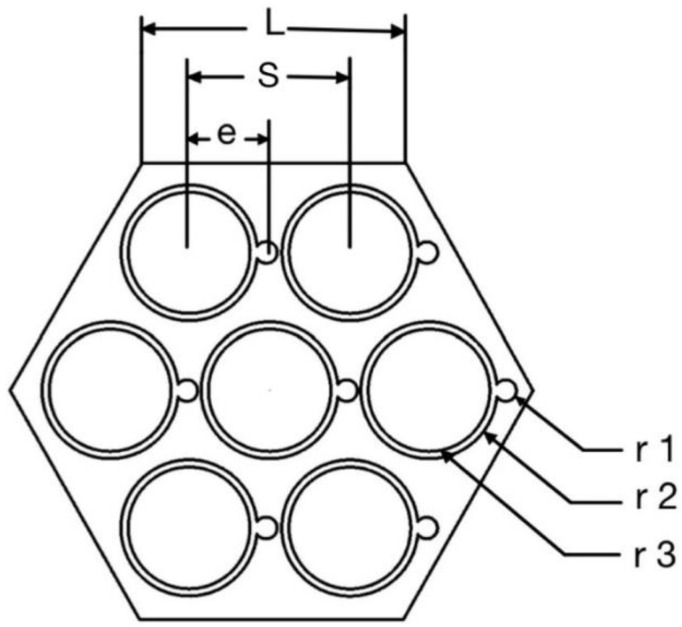
The planform of the flow region and fuel assembly.

Figure 1 shows the total geometry model including fluid domain, wire-wrapped winding and fuel bundle. Figure 2 shows the wire-wrapped fuel bundle. Figure 3 shows the planform of the flow region and fuel assembly. The geometries of rod and wrapping wire are the same as reference [14]. The geometry parameters are listed in Table 1. The wires are embedded into the claddings with 3.375 mm from the center of the rod. For the fuel rod bundle, double helical pitch length was selected in calculation. In order to decrease the influence of boundary conditions for inlet and outlet, 50 mm and 80 mm flow lengths are added at inlet and outlet, separately, as shown in Figure 1. Hence, the inlet velocity condition and pressure outlet condition can be imposed on both ends. The walls of wire, cladding and duct are defined as no slip boundary conditions. Volume heat resource (1.3974 × 10^9^ W/m^3^) is used only in the rod bundle (Table 2).

Fluid was regarded as a Newtonian and incompressible liquid; its physical properties change with temperature. The fitting polynomial of the properties are as follows [15]:

(5) ρ=1016.82−0.239T

(6) $$C_{p}=1629.1-0.83267T+0.00046208T^{2}$$

(7) λ=105.1843−0.049T

(8) $$\mu =0.00385-1.66448\times 10^{-5}T+3.00411\times 10^{-8}T^{2}-2.48661\times 10^{-11}T^{3}+7.8085\times 10^{-15}T^{4}$$

The applicable temperature range of the above fitting formulas was 373.15 K to 1073.15 K. In the simulation of flow, the reference temperature was selected as 645 K. The materials of cladding and fuel rod bundle were stainless steel Ti316 and UO_2_, separately. Their physical properties are listed in Table 3.

### 2.2. Meshing

**Figure 4:**
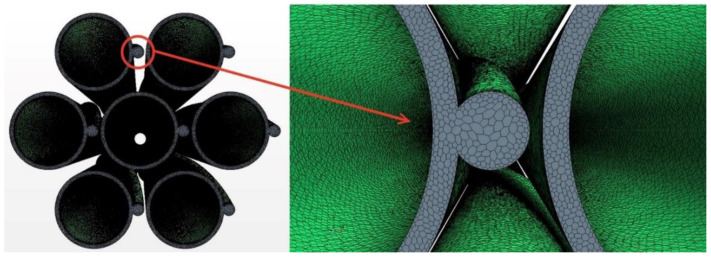
The meshes between wire and rod.

**Figure 5:**
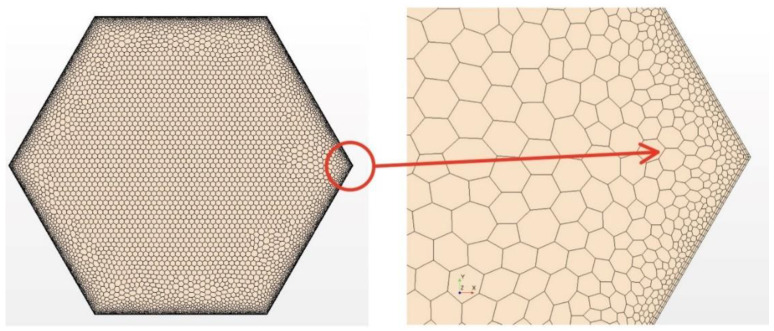
The meshes of inlet section and the corner.

The mesh was created by Star CCM+ [13]. The prism layer mesh was employed to generate the grids in the near-wall region. Meanwhile, a polyhedral mesh generator and surface reconstruction were applied to get high-quality grids [13]. Figure 4 and Figure 5 show the mesh model and local details,$\;Y^{+}$ is kept around 1 at these areas and other near-wall region to meet the wall function requirements. It can be found that near the wall, not only the fluid region but also the cladding region the meshes are refined. In traditional calculation, the meshes for thermal conductivity are coarse. Here near the heat transfer wall, the mesh densities in the fluid and solid domains are comparable; this idea comes from the critical heat flux numerical simulation [16]. Due to the extreme inhomogeneity of wrapping wire and fuel rod circumferential heat transfer [14], more meshes are used to resolve these local temperature fields. The meshes applied in this article are the same as the densest mesh case in [14] by Wang et al., which has been verified for grid independence. The total mesh number is 8,357,610, with 261,048 in the fuel rod bundle, 2,637,278 in the wrapping wire and 5,459,284 in the fluid region. Using these parameters, the phenomenon worth mentioning below were discovered.

**Figure 6:**
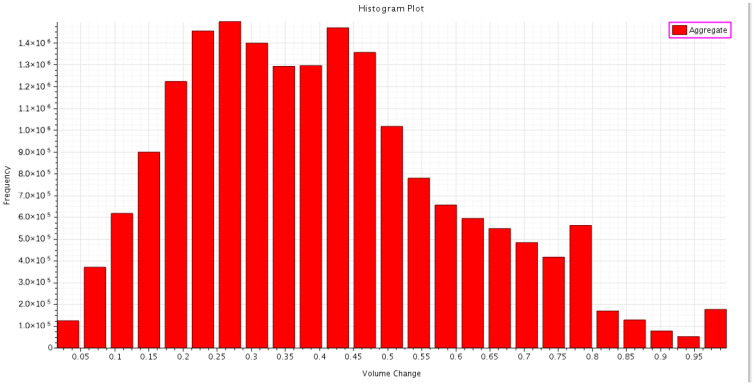
Volume change of mesh unit.

Volume change is a very important value which can measure the quality of the mesh unit in Star CCM+. If the value is 1.0, it means that the mesh has a larger or equal volume than its neighbors, and if the value is 0.01 or less, it is considered a poor mesh quality [13]. As shown in Figure 6, 92.530% of the grids are in the interval 0.1 to 1, and 7.470% of the grids are in the interval 0.01 to 0.1. Therefore, under the evaluation criteria of Star CCM+, the mesh quality is guaranteed.

### 2.3. Reversed Heat Fluxes in Fluid Flow

**Figure 7:**
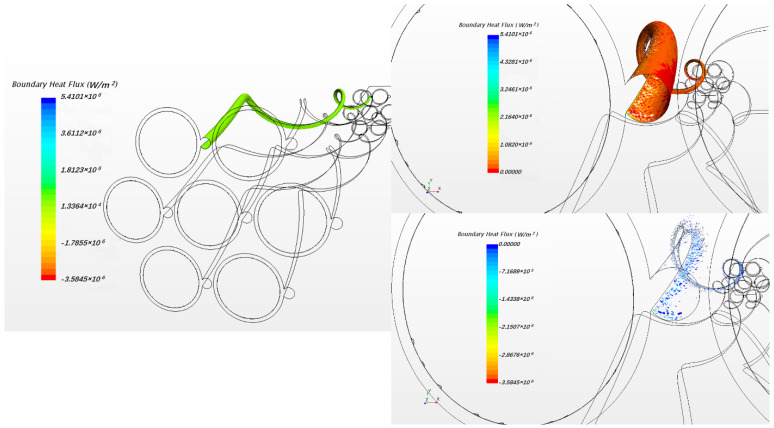
The heat flux on one wire surface with the positive and reversed heat fluxes.

**Figure 8:**
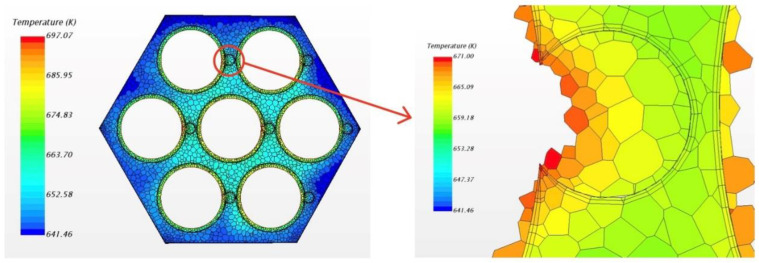
The temperature field of cross section and local magnification around wrapping wire.

In this study, the fluid case was calculated by commercial CFD codes of Star CCM+ on a 32-core computer. The convergence criterion was set to $10^{-5}$ and it took around 10 h to reach the criteria by 32-core parallel calculation. As per usual understanding, the heat flux will pass through the cladding and wrapping wire into fluid. However, in the result of the present steady simulation, we found that heat fluxes exist from fluid back to wire in the junction area between wire and cladding, as Figure 6 shows. This phenomenon exists in all the 7 pins for the whole wire, but the maximum value of heat flux in each wire is different, probably owing to flow rotation. For easy observation, Figure 7 only shows the phenomenon of one pin; the picture on the left shows the overall heat fluxes in the wrapped wire and the picture below in the right hand shows reversed heat fluxes. The average value of the reversed heat fluxes is about $8\times 10^{5}\;W/m^{2}$ which is pretty close to the normal fluxes in the picture in the upper right of Figure 7, and cannot be a numerical error. Figure 8 shows the temperature field of the vertical section plane (the middle part of the bundle) of flow direction. Near the junction there are some areas where the temperatures of fluid are higher than those in the wire or cladding region. This may explain how the reversed heat fluxes are generated.

**Figure 9:**
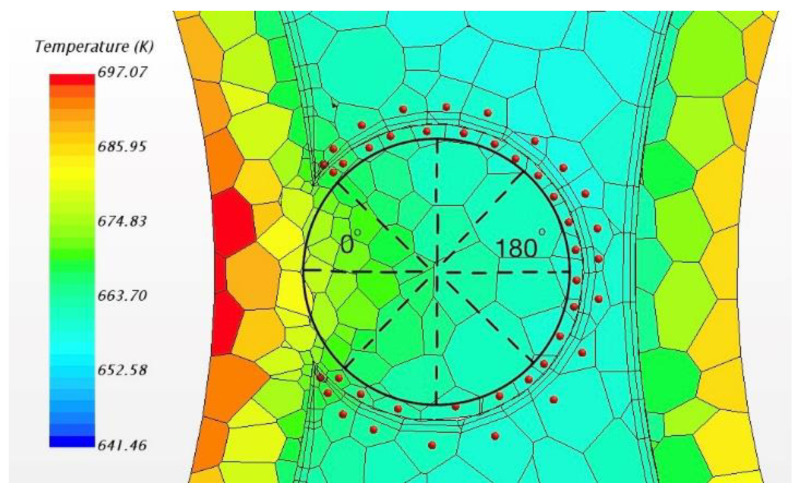
The direction of the angle and temperature spots.

**Figure 10:**
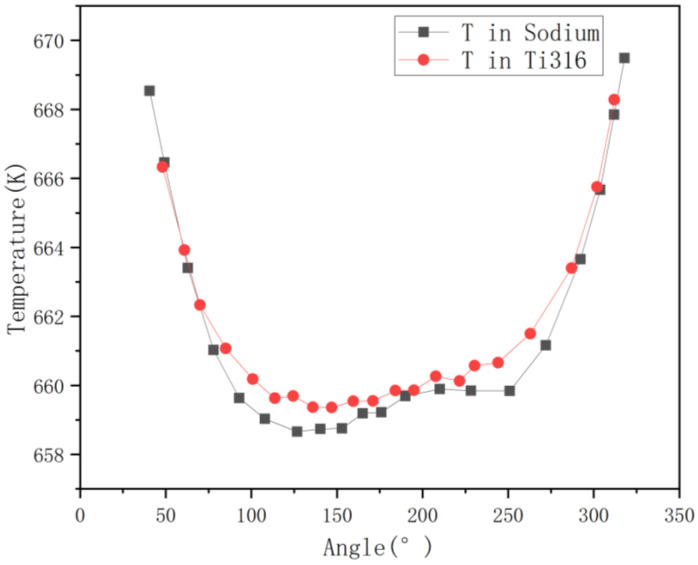
Temperature curves of sodium and wrapping wire around the wrapping wire.

Figure 9 shows the direction of the angle and temperature spots, which will be used in the next analysis. Circumferential temperature distributions of wire and sodium can be seen in Figure 10. As the figure shows, the maximum temperature is located in sodium compared with the wrapping wire. While the angle is around 50 degrees, the temperature in sodium is higher than that in the wire. Within a 20 degrees angle difference, the temperature difference can even reach almost 6 K, which is a very impressive value, and that is why the reversed heat fluxes can reach such a high level. Hence, the heat transfer from sodium to wrapping wire becomes a necessity. Furthermore, in flow condition, the flow near the junction is close to the wall so the velocity is very low, which can lead to insufficient heat convection. Heat conduction probably plays an important role here.

## 3. Solid Model

**Figure 11:**
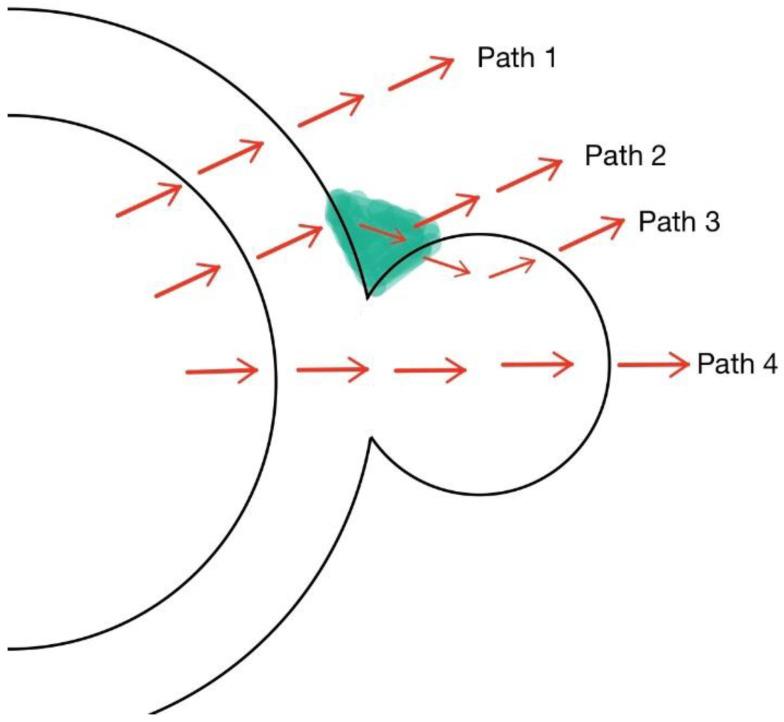
Potential paths of heat fluxes.

**Figure 12:**
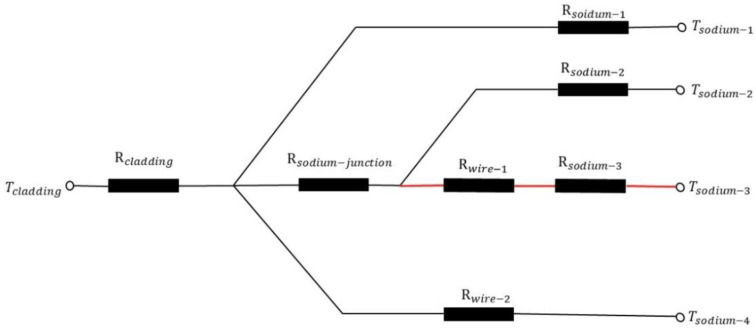
Thermal resistance for each path.

There are many factors that will have influence on heat exchange in the simulation of fluid and as the above shows, the phenomenon is not very obvious in fluid calculation. The velocity of flow in the junction is much smaller than in the main flow so the heat conduction plays a major part of heat transfer in the junction. After considering these, a solid heat conduction model is proposed, in which only the fluid region is turned into a solid one, to prove the existence of this phenomenon. In the solid model, the properties of sodium are kept the same as fluid. There are four potential paths that the heat flux may pass through the wire and cladding to the sodium as Figure 11 shows. Figure 12 shows the thermal resistance of the four paths. If we fix attention on the junction, it can be found that there might be two directions for the flux to pass as path 2 and path 3 show. It is pretty hard to tell in which path the thermal resistance is smaller, but we believe that the heat flux through path 3 produces this special phenomenon.

Furthermore, since the thermal resistance from flow to wrapping wire in fluid is obviously smaller than that in solid, the tendency of reversed heat fluxes in fluid is stronger than it in the solid. Therefore, if reversed heat fluxes still exist in the solid model, they must also exist in the fluid model.

### 3.1. Geometry Model of the Solid

**Figure 13:**
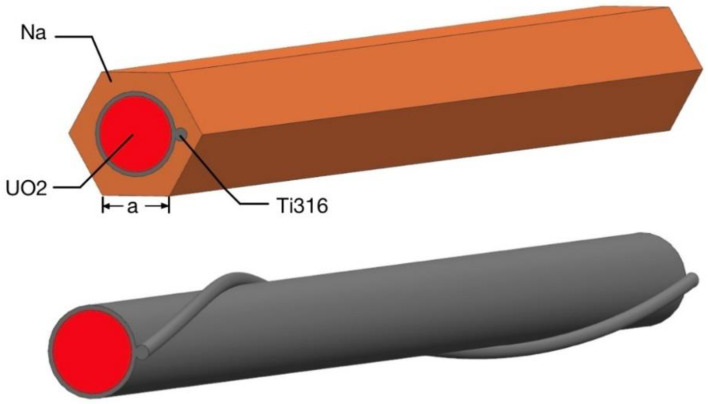
Shape and material of the solid model.

In order to reduce the amount of calculation, a one-pin model was selected as simplification. Geometric dimensions of rod, winding and wire are the same as fluid model shown in Table 1. The external side length (“a”) is 5 mm. Figure 13 shows the shape and materials of solid model and Table 4 shows the boundary conditions. The physical properties of rod, wire cladding and external solid region(sodium) are listed in Table 5. Thus, we can now proceed to use the solid model for investigation of some interesting factors related to the phenomenon described.

### 3.2. Meshing

**Figure 14:**
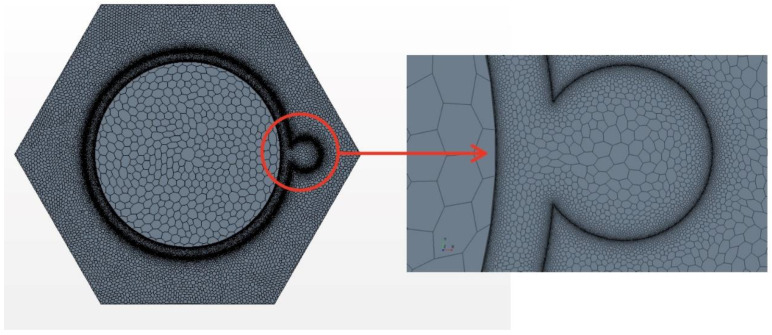
The mesh of solid model and local detail view profile.

Surface reconstruction and repair technology and polyhedral mesh generator of star CCM+ are applied to generate high-quality grids. Curve control near the junction is used to generate denser grids so that the shape of the junction is closer to reality and the accuracy of simulation can be higher. Furthermore, it can get more accurate values such as temperature, fluxes, and so on. Figure 14 shows the grids and details of typical mesh near the helical wire spacer. Table 6 shows the detailed information about the mesh.

### 3.3. Sensitivity Analysis of Mesh

Mesh resolution can have a great influence on the simulation accuracy. Thus, to evaluate the dependency of simulation accuracy on the mesh resolution, three different mesh types were considered in the validation calculation. Base size is an important value which can decide the scale and number of the grids generated by Star CCM+. With a larger base size, less mesh is expected. In the three cases, mesh base sizes in region of Na and wire cladding are respectively set to 0.1 mm, 0.12 mm and 0.16 mm, while base size of rod is still 0.26 mm. Mesh numbers for each case are shown in Table 7. For the solid cases, the convergence criterion is that the scaled residual falls below $10^{-13}$ and it took about 40 min to get the results while using 28 threads for parallel computing. The time of calculation was only 1/15 that of the fluid calculation. Hence, the solid model can greatly decrease the costs and save time.

**Figure 15:**
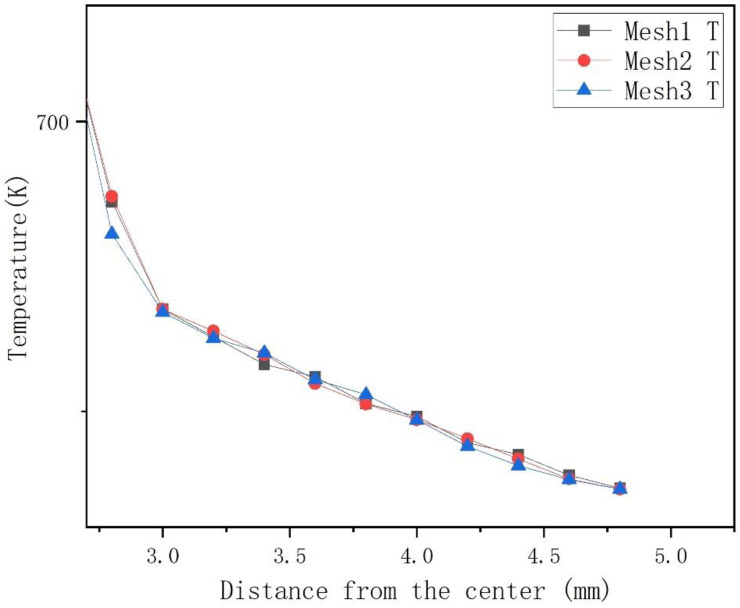
Radial temperature profiles for different mesh.

To prove the calculation results are independent of mesh resolution, 15 points with radially equidistant interval of 2 mm were selected in the section perpendicular to the pitch direction, at the middle length of fuel rod. Figure 15 shows temperatures at those points. From the three temperature lines in Figure 15, it is easy to see that the temperature distributions of case 1 and case 2 are close. Therefore, base size in case 1 was selected in subsequent calculations.

### 3.4. Simulation Results of Solid Model

**Figure 16:**
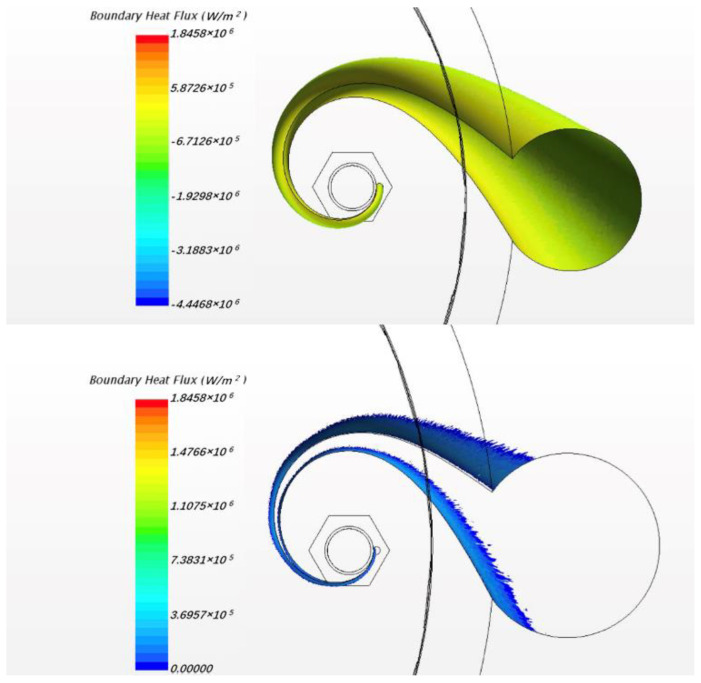
The schematic of heat flux along the wrapping wire surface under solid model.

**Figure 17:**
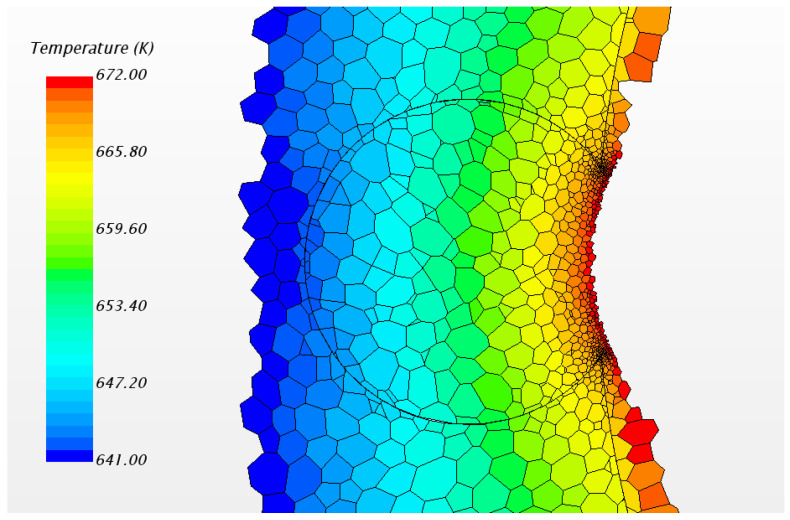
Local temperature field of wrapping wire.

Figure 16 shows the phenomenon of reversed heat flux for solid model. Here positive heat flux values means that the direction of the flux is from solid sodium to wire and so the second picture in Figure 16 indicates the location and magnitude of the reversed heat flux. The maximum value exceeds $1.8\;\mathrm{MW}/m^{3}$ which is about 40% of the normal fluxes, very notable. Obviously, it is more pronounced than in the flow condition. The local temperature field is shown in Figure 17.

**Figure 18:**
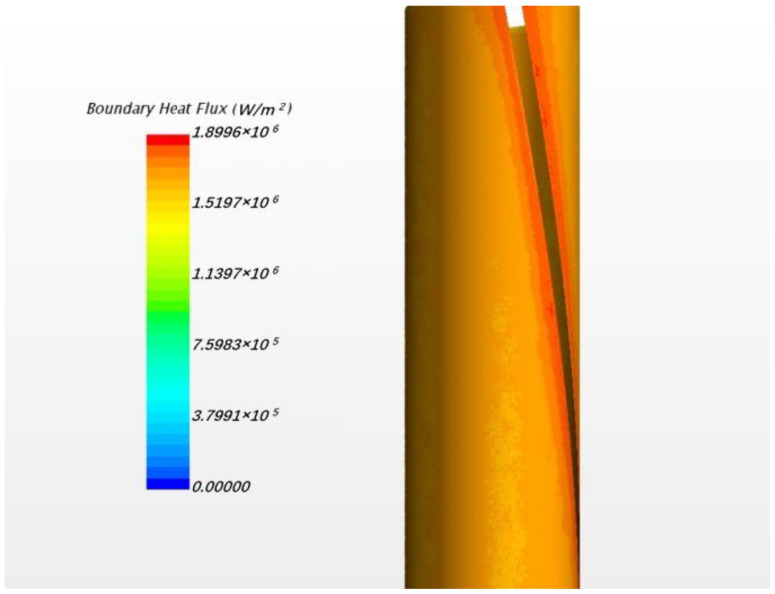
The surface heat flux of the rod.

In Figure 17, it can be found that, near the junction, there is a decrease of temperature from sodium to wrapping wire. Although the temperature difference is not obvious, the fluxes still cannot be ignored. It is about the order of $2\times 10^{5}$ in main area of reversed fluxes while the normal fluxes in wrapping wire is about $4\times 10^{5}\;W/m^{2}$. Meanwhile, the structure of the wrapping wire also has a significant effect on the heat flux distribution of the surface of cladding. Figure 18 shows that the fluxes near the contact area between wrapping wire and cladding is obviously higher than other places, meaning that it is unreasonable to set boundary conditions for surface heat fluxes and to ignore heat transfer in fuel rod and wire cladding as a simplification, which are often applied in previous numerical simulations of fluid. Because the thermal resistance passing through the wrapping wire is large, the heat passing through the wrapping wire originally will go through the rod surface near the junction where the heat resistance is smaller, and so the heat flux on the rod surface next to the wrapping wire increases. In fluid calculation, similar results can also be found. In addition to the similar cause of heat flux redistribution caused by thermal resistance, the following situation may also be responsible. Due to the presence of wrapping wire, although the flow rate will be reduced due to narrow size, the secondary flow will impact this position directly, so the heat transfer will be increased [14]. Thus, more heat is concentrated in this narrow space, causing the sodium temperature to rise even higher than that of the wire. Thus, the phenomenon of reverse heat transfer occurs. The influence of some obvious factors on this phenomenon will be discussed in detail, hoping to provide guidance for CFD calculation.

## 4. Factors Influencing the Phenomenon

### 4.1. Embedding Depth of Wrapping Wire

The contact between wrapping wire and cladding has a significant effect on the calculation results because the geometry at the junction will change with it. The embedding depth is often described by the distance from wire to rod (“e”) as defined in Table 1. Three different embedding depths are shown in Table 8. Besides the embedded contacts, there is also another contact method in which the wrapped wire is separated from the cladding with a short distance 0.005 mm, as in case 4.

**Figure 19:**
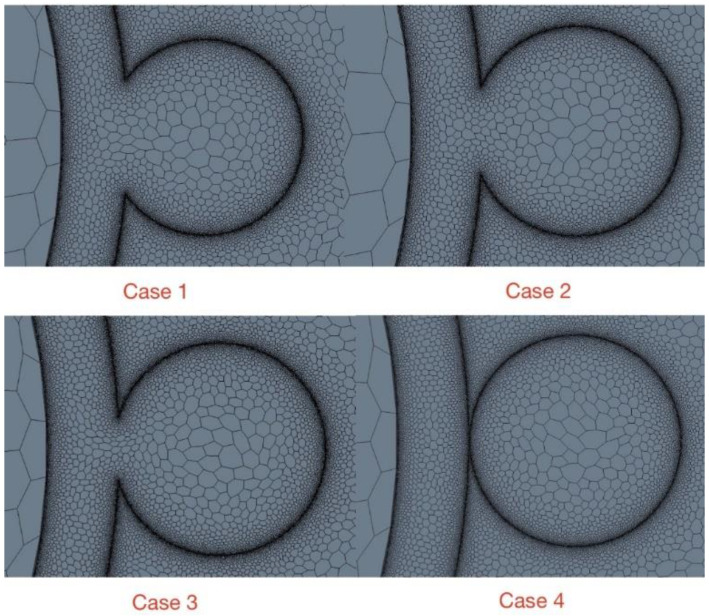
Four embedding depths.

From case 1 to case 3, the embedding depth gradually decreases which means the distance “e” increases and the sharp corner at the junction gets more distinct. In this way the situation is approaching engineering practice. Figure 19 shows the details of four embedding depths which decrease from case 1 to case 4.

**Figure 20:**
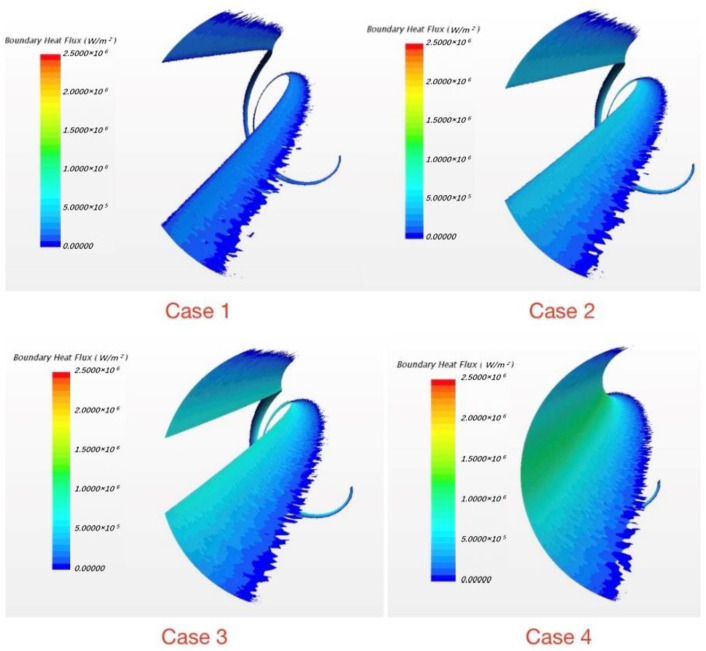
The reversed heat flux varies with embedding depth.

As shown in Figure 20, with the embedding depth decreasing, the magnitude of reversed heat fluxes increases. The reversed heat flux region in case 3 and case 4 are almost twice as large as it in case 1. Furthermore, areas where the phenomenon exists are also gradually expanding; in case 4, the area of reversed heat fluxes is about half of the area of wrapping wire. Therefore, the embedding depth can have a great influence on this phenomenon because it can change the thermal resistance at the junction while geometry varies. Hence, in flow analysis, the commonly used wrapping wire embedding method may weaken the inverse heat transfer phenomenon. This may be why the phenomenon has not been found and studied before.

### 4.2. Calculation Domain

Since the solid model is a simplification of the flow model, the size of calculation domain may have an effect on the results. Therefore, three cases in which the value of side length “a” in Figure 13 varies were calculated to find out those influences. The side lengths for the three cases were 5 mm, 6 mm and 7 mm, respectively.

**Figure 21:**
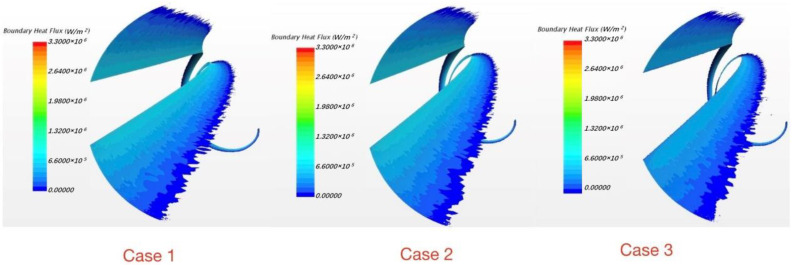
The reversed heat flux varies with calculation domain.

Considering that for smaller embedding depth, the phenomenon is more obvious and easily observed, the embedding depth “e” is set to 3.45 mm, and in Figure 21 the three figures from left to right are case 1, case 2 and case 3. As for the maximum value of reversed heat fluxes in the three cases, in case 1, it is $8.0\times 10^{5}\;W/m^{2}$ and in case 2 it is $7.5\times 10^{5}\;W/m^{2}$, while in case 3 it is $5.2\times 10^{5}\;W/m^{2}$. The maximum value is near the junction in all cases, and the phenomenon is obviously stronger in case 1 than in the other two cases. Besides the maximum value, the size of the area for reversed heat fluxes also changes with the side length; the area ratio for these three cases is around 11:9:8. Therefore, the conclusion is that the reversed fluxes will get weaker if the areas where the fluxes exist get smaller and the calculation domain increases.

### 4.3. Physical Properties

In SFR the coolant is sodium and material of cladding and wrapping wire is Ti316 alloy. Because thermal conductivities vary in different materials, which directly affects the thermal resistance, physical properties have a significant impact on such phenomenon. To figure out how the physical properties affect the phenomenon, four cases were calculated for comparison and are shown in Table 9. As an original comparison, the embedding depth is set to 3.45 mm and the side length “a” is 5 mm in case 0. All the following cases are derived from case 0.

**Figure 22:**
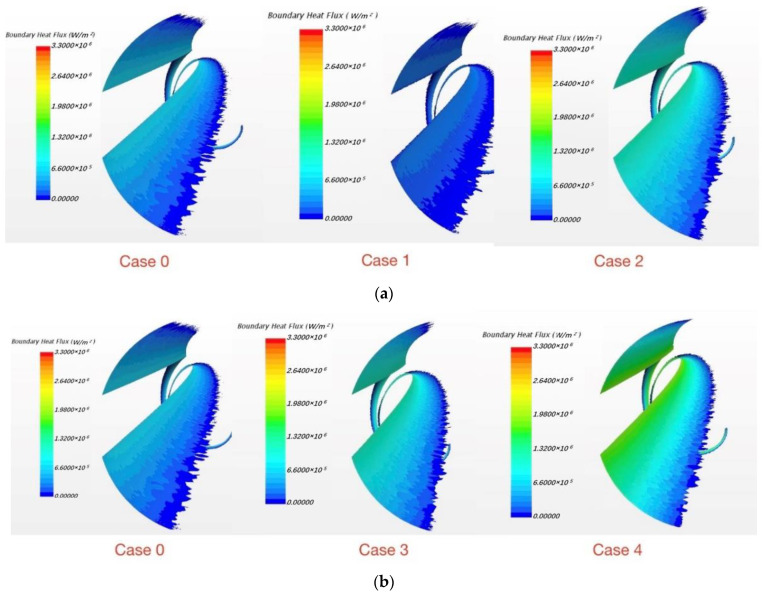
Physical property variation: (**a**) Thermal conductivity changes of sodium; (**b**) thermal conductivity changes of Ti316 alloy.

**Figure 23:**
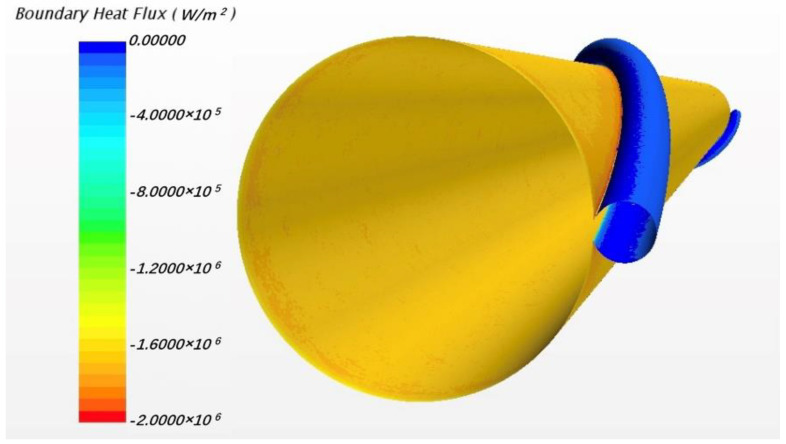
Heat fluxes at eight times thermal conductivity of sodium.

In Case 1, conductivity of sodium doubles as before which means its thermal resistance becomes half of the previous value. Thus, heat transfer is better in the coolant, and heat fluxes only transferred by coolant are more than going back through the wrapping wire. In this way, the phenomenon of reversed fluxes become weaker. Under the same logic, the phenomenon gets stronger with the decrease of thermal conductivity of sodium as seen in Figure 22a. In the same way, increasing the thermal conductivity of the material of the wrapping wire can strengthen the phenomenon as Figure 22b shows. The result of the case for original comparison is case 0 in Figure 22a,b. Hence, under flow condition, due to convection it looks like the thermal conductivity of sodium is increased. Then the reversed heat flux phenomenon is reduced. However, because of the narrow junction, the convection heat transfer is very weak; thus, heat conduction is actually the most important factor. Moreover, we boldly increased the thermal conductivity to find out that when the thermal conductivity is eight times its actual value, the phenomenon will disappear, as shown in Figure 23. It is believed that with such a large thermal conductivity, almost all the heat fluxes are passed by path 1 in Figure 11. Under such condition, there is almost no heat flux transferred by the wrapping wire. However, in engineering practice, a coolant with such a large thermal conductivity does not exist. Thus, this phenomenon has a large possibility to exist in practice.

## 5. Conclusions

As described in this article, a reversed heat flux phenomenon near the junction between wrapping wire and cladding was discovered by accident in the simulation of a sodium-cooled 7-pin wire-wrapped rod bundle model. The same phenomenon in an imaginary solid thermal conductivity model confirms that it does exist. Some factors have been considered and researched. The main conclusions are listed as follows:

With an increase in embedding depth, the phenomenon of reversed heat flux is enhanced and the average value of the reversed heat flux gets larger. The traditional embedding method in CFD may not get this phenomenon because it may obtain an incorrect local temperature field.

Thermal conductivities of coolant and wire cladding both have great influence on the reversed heat flux by the thermal resistance on the path of reversed flux. Increasing the thermal conductivity of the wrapping wire or decreasing the thermal conductivity of the coolant can significantly enhance this phenomenon. When the thermal conductivity of sodium is eight times its actual value, the phenomenon will disappear.

Finally, in previous simulation research of flow, regarding the walls of wrapping wire as adiabatic and choosing homogeneous surface heat fluxes boundary condition are often adopted to reduce computation quantity. However, according to present results, it is obvious that such simplifications are unreasonable to some extent. Moreover, though high temperature near the junction between the wrapping wire and cladding has already been noticed, it is often explained by low coolant velocity near the wall, but the results in this article may provide another explanation. Furthermore, the work in this article can provide reference values for future flow calculation, especially in grid generation and treatment of the junction between the wrapping wire and the fuel rod.

## Figures and Tables

**Table 1:** Geometric parameters.

Parameter	Value
Helical pitch	100 mm
Duct side length (L)	11.58 mm
Pin radius (r1)	2.58 mm
Cladding radius (r2)	3 mm
Wire radius (r3)	0.95 mm
Pin pitch (S)	7 mm
Distance from rod to wire (e)	3.375 mm
Pitch number	2
Number of pins	7

**Table 2:** Boundary conditions.

Boundary	Conditions
Inlet	Mass flow inlet
outlet	Pressure outlet
Inlet temperature	633.15 K
Duct walls	Adiabatic
All the walls	No slip
Mass flow (Q)	1.0 kg/s
Heat source	Volume source in rod bundles
Volume source	1.3974 × 10^9^ W/m^3^
Reynolds number	60,285

**Table 3:** Physical properties.

	UO_2_	Ti316
Density	$9560\;\mathrm{kg}/m^{3}$	$7995\;\mathrm{kg}/m^{3}$
Thermal conductivity	4.4709 W/m·K	21.8 W/m·K
Specific heat	316.6071 J/kg·K	560 J/kg·K

**Table 4:** Boundary conditions.

Boundary	Conditions
Duct walls	Temperature
Wall temperature	633.15 K
Heat source	Volume source in rod
Volume source	$1.3974\times 10^{9}\;W/m^{3}$

**Table 5:** Physical properties.

	Na	UO_2_	Ti316
Density	$862.665\;\mathrm{kg}/m^{3}$	$9560\;\mathrm{kg}/m^{3}$	$7995\;\mathrm{kg}/m^{3}$
Thermal conductivity	73.5793 W/m·K	4.4709 W/m·K	21.8 W/m·K
Specific heat	1284.2647 J/kg·K	316.6071 J/kg·K	560 J/kg·K

**Table 6:** Details of solid model grids.

Region	Base Size	Number of Grids
Na	0.1 mm	4,614,753
Wires and cladding	0.1 mm	10,115,338
Rod	0.26 mm	106,596
Total		14,836,687

**Table 7:** Mesh number.

	Base Size	Rod	Wire Cladding	Na	Total
Case 1	0.1 mm	106,596	10,115,338	4,614,753	14,836,687
Case 2	0.12 mm	106,596	7,126,721	3,560,360	10,793,677
Case 3	0.16 mm	106,596	4,086,034	2,038,983	6,231,613

**Table 8:** Embedding depths.

No.	Distance from Wrapping Wire to Rod “e”
Case 1	3.375 mm
Case 2	3.425 mm
Case 3	3.475 mm
Case 4	3.480 mm

**Table 9:** Thermal conductivities of sodium and Ti316 for five cases.

	Na	Ti316
Case 0	$\lambda_{a}$	$\lambda_{b}$
Case 1	$2\lambda_{a}$	$\lambda_{b}$
Case 2	$0.5\lambda_{a}$	$\lambda_{b}$
Case 3	$\lambda_{a}$	$2\lambda_{b}$
Case 4	$\lambda_{a}$	$5\lambda_{b}$

Note: $\lambda_{a}$ and $\lambda_{b}$ are conductivities of sodium and Ti316 alloy.

## Data Availability

The data presented in this study are available on request from the correspond author. The data are not publicly available due to privacy.

## Nomenclature

$Q_{v}$	Calorific value per unit volume ($W/m^{3}$)
$C_{p}$	Specific heat capacity (J/kg/K)
Λ	Thermal conductivity of fuel rod (W/m/K)
T	Temperature (K)
Ρ	Density of fuel rod ($\mathrm{kg}/m^{3}$)
T	Time (s)
R	Radius of any point in fuel rod (mm)
$R_{f}$	Radius of fuel rod (mm)
$T_{s}$	Temperature at the edge of fuel rod (K)
Μ	Molecular viscosity (kg/m/s)
$U_{i},U_{j}$	Cartesian velocity components (m/s)
$x_{i},x_{j}$	Cartesian coordinates (m)
$Pr_{\;}$	Prandtl number
$Pr_{t}$	Turbulent Prandtl number
$Pe_{t}$	Turbulent Peclet number
P	Pressure (Pa)
